# LRP6 Receptor Plays Essential Functions in Development and Human Diseases

**DOI:** 10.3390/genes13010120

**Published:** 2022-01-10

**Authors:** Abdulmajeed Fahad Alrefaei, Muhammad Abu-Elmagd

**Affiliations:** 1 Department of Biology/Genetic and Molecular Biology Central Laboratory (GMCL), Jamoum University College, Umm Al-Qura University, Makkah 2203, Saudi Arabia; 2 Center of Excellence in Genomic Medicine Research, King Abdulaziz University, Jeddah 80216, Saudi Arabia; mabuelmagd@kau.edu.sa

**Keywords:** Lrp6, LDL, Wnt/β-catenin signalling, gene expression and function, development, human disease, genetic disorder

## Abstract

LRP6 is a member of the low-density lipoprotein receptor superfamily of cell-surface receptors. It is required for the activation of the Wnt/β-catenin signalling pathway. LRP6 is detected in different tissue types and is involved in numerous biological activities such as cell proliferation, specification, metastatic cancer, and embryonic development. LRP6 is essential for the proper development of different organs in vertebrates, such as *Xenopus laevis*, chickens, and mice. In human, LRP6 overexpression and mutations have been reported in multiple complex diseases including hypertension, atherosclerosis, and cancers. Clinical studies have shown that LRP6 is involved in various kinds of cancer, such as bladder and breast cancer. Therefore, in this review, we focus on the structure of LRP6 and its interactions with Wnt inhibitors (DKK1, SOST). We also discuss the expression of LRP6 in different model systems, with emphasis on its function in development and human diseases.

## 1. Overview of Wnt/LRP6 Signalling

LRP6 is a member of the low-density lipoprotein (LDL) receptor superfamily of cell-surface receptors. It is required for the activation of the Wnt/β-catenin signalling pathway [1]. It is detected in different tissue types and involved in numerous biological activities such as cell proliferation, specification, migration, metastatic cancer, and embryonic development [2]. Biochemical and genetic studies have discovered that members of the LDL receptor-related (LRP) family, specifically LRP5 and LRP6 in vertebrates, are coreceptors of the canonical Wnt signalling pathway [3,4]. LRP6 binds to Wnt and Frizzleds (Fzds) in a ternary complex.

LRP6, together with Fzds, plays an essential role in the activation of the canonical Wnt/β-catenin signalling pathway as a Wnt coreceptor [4,5]. Wnts are a family of secreted molecules that regulate a wide variety of biological processes such as embryonic development [2]. The canonical Wnt signalling pathway (Wnt/β-catenin) consists of several components, including 19 Wnt ligands, 10 FZD receptors, and FZD coreceptors such as LRP5/6 [2,6]. In addition, LRP6 is regulated by inhibitory proteins such as Dkk1, Sclerostin (Sost), Wise, and ZNRF3. These Wnt antagonists modulate LRP6 activation by Wnt members.

**Figure 1:**
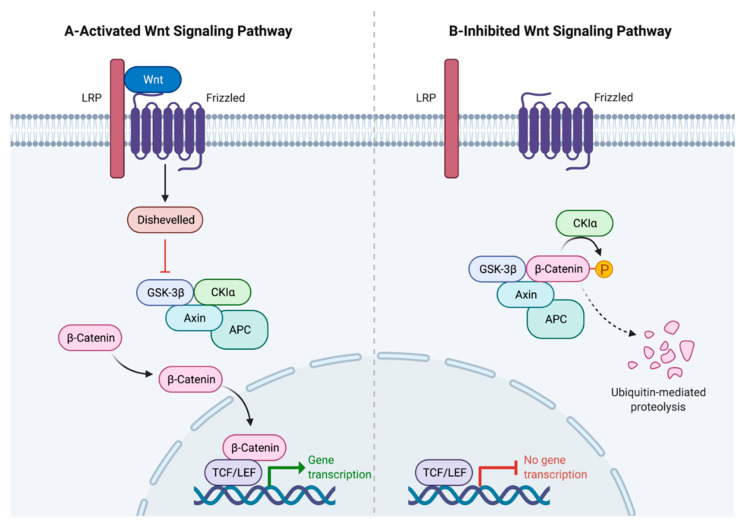
Wnt/β-catenin signalling pathways. (**A**) Canonical Wnt signalling. Wnt ligands bind to FZD/LRP5/6 receptors, which leads to Dishevelled (Dvl) activation. This is followed by inhibition of the destruction complex Axin/APC/GSK-3β/CKI, and the degradation of β-catenin is stopped, resulting in the accumulation and stabilisation of β-catenin in the cytoplasm. After accumulation, β-catenin enters the nucleus and interacts with LEF/TCF DNA-binding transcription factors. This results in the activation of Wnt target genes. (**B**) Canonical Wnt signalling (without Wnt binding). The destruction complex mediates β-catenin degradation through proteasomal machinery, leading to the association of LEF/TCF transcription factors with transcriptional corepressors in the nucleus, which leads to the repression of Wnt target genes.

The stability of β-catenin relies on the presence of Wnt ligands [2]. In the absence of Wnt stimulation, β-catenin is degraded by the destruction complex Axin/APC/GSK-3β/CKI (Figure 1B). The binding of Wnt ligands to complex FZD/LRP5/6 receptors leads to the recruitment of Dishevelled (DVL) and Axin via the intracellular domains of FZD and LRP5/6. Subsequently, LRP6 signalosomes are formed, and LRP6 phosphorylation occurs via CK1γ and GSK3β, resulting in the inhibition of β-catenin phosphorylation [4,7]. The accumulation and stabilisation of β-catenin is then increased in the cytoplasm. Consequently, β-catenin enters the nucleus and interacts with LEF/TCF DNA-binding transcription factors. This results in the activation of Wnt target genes (Figure 1A) [8].

It is clear that LRP6 is required for Wnt signalling activation. Genetic and biochemical experiments have revealed that LRP6 plays an essential role in development, cell proliferation, and human diseases. Importantly, LRP6 overexpression and mutation has been detected in several types of disease, including hypertension, atherosclerosis, hypercholesterolemia, and cancer. Therefore, we focus here on the structure and expression of LRP6, with emphasis on its function in development and human diseases.

## 2. LRP6 Structure and Homology

**Figure 2:**
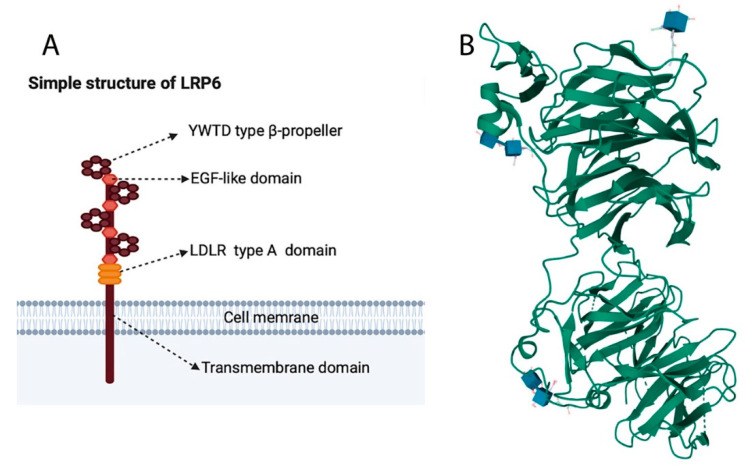
Structure of LRP6. (**A**) LRP6 consists of four YWTD β-propeller domains, each of which is followed by an EGF-like domain, and this is followed by three LDLR type A repeats, a transmembrane domain, and a short intracellular domain. (**B**) This structure was modelled using PDB protein databank software with ID number 3S94 according to the crystal structure of LRP6–E1E2, with green representing YWTD domains. This 3D structure of human LRP6 protein covers 37% of the sequence (585 residues from 21 to 628) with 100% confidence (Supplementary Figure S1), and it contains the binding site of Wnt members and some Wnt inhibitors such as DKK1.

Lrp6 is a single-pass transmembrane protein involved in the activation of Wnt signalling pathways [1,2]. The human LRP6 has 23 exons, is found in chromosome 12p 13.2, and contains 1613 amino acids [9]. LRP6 consists of four YWTD β-propeller domains, each of which is followed by an EGF-like domain, and this is followed by three LDLR type A repeats, a transmembrane domain, and a short intracellular domain [10] (Figure 2A). It is structurally related to LRP5, and they share almost 71% homology at the nucleotide level [2,5].

In addition, the human Lrp6 crystal structure protein has been characterised using electron microscopy (EM) and other molecular/bioinformatic tools [10,11,12,13,14,15] (Figure 2B). LRP6-E1E2 or LRP6-E3E4 proteins were purified and concentrated for crystallisation [10]. The crystal structure of the extracellular domain of Lrp6–E1E2 was revealed; E1 is the first fragment, including a YWTD-type β-propeller and an EGF pair of Lrp6, while E2 is the second fragment, containing a YWTD-type β-propeller and an EGF pair. This crystal structure shows that the propeller axes of the two YWTD propellers are almost parallel, and the two EGF domains adhere to the bottom surface of the previous YWTD propellers in a manner that is nearly identical to that of the YWTD–EGF pair in LDLR [10]. Furthermore, the crystal structures show that the top surfaces of LRP6-E2 and LRP6-E3 are remarkably different, whereas LRP6-E1 and LRP6-E3 have great similarity in terms of the top surfaces. These structural details improve our understanding of LRP6 protein function and signal interactions, which could enhance its targeting for therapeutic purposes. For instance, several Wnts, such as Wnt1, Wnt2, Wnt2b, Wnt6, and Wnt8a, interact with the Lrp6–E1E2 domain, whereas other Wnt ligands prefer binding to other domains of LRP6 [2,16]. This binding preference (Wnts-LRP6) could help researchers in the development of drug specificity or uncovering molecular mechanisms to target Lrp6 or treat Wnt-related diseases.

Understanding LRP6’s structure and interactions with Wnt inhibitors such as DKK1 [17,18,19] and SOST [13,20] is essential to treating human diseases and the development of drugs with high selectivity [20,21,22,23,24,25]. In this regard, the structure of the LRP6 and DKK1c (C-terminal Cys-rich domain) protein complex reveals that DKK1c can interact with both LRP6 fragments (E1E2–E3E4), as determined by an α technology binding assay [10]. Therefore, this interaction enhances our knowledge in terms of how DKK1 regulates LRP6 and Wnt signalling and could be employed in drug development. Recently, the crystal structure of SOST was investigated, because it is an antagonist of Wnt signalling and interacts with LRP6. Thus, the crystal structure of LRP6 E1E2 and SOSTtr177 (a C-terminal 37-amino-acid truncation mutant of SOST) led to the discovery of an additional binding site between the LRP6 E2 domain and the C-terminus of SOST [26]. This finding was validated in vivo using *Xenopus laevis* embryos and via in vitro binding. These results advance our understanding of how SOST inhibits Wnt signalling by antagonising LRP6 [26]. Importantly, this inhibitory molecular mechanism could be used as a therapeutic target for osteoporosis and other diseases [20]. The availability of the crystal structures of hLRP6, DKK1c, and SOSTtr177 will help researchers to explore the molecular mechanisms of Wnt activators (receptor oligomerisation) and inhibitors.

**Figure 3:**
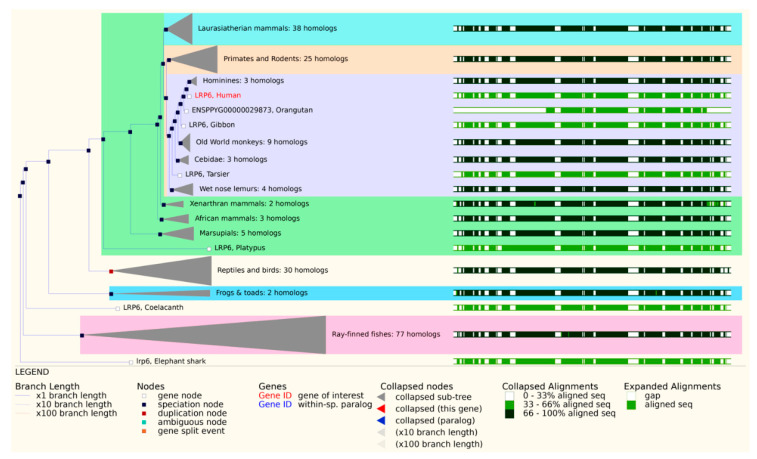
Gene homology tree showing human LRP6 in comparison to LRP6 homologs in other species in the animal kingdom (Ensembl reference number: GeneTree ENSGT00940000158990).

Furthermore, Lrp6 is an evolutionarily conserved gene among all animals [4] (Figure 3). For example, Arrow was cloned as a member of the LDLR family in Drosophila, and it is homologous to Lrp5 and Lrp6 in vertebrates [27,28,29]. Arrow shows 45% similarity with Lrp5 and Lrp6 proteins [4]. In addition, Arrow and Lrp6 mutants share similar phenotypes with Wg/Wnt mutants [3,29]. Furthermore, human and mouse LRP6 show 98% similarity at the protein sequence level [27]. Additionally, HLRP6 shares more than 93% identity in its amino acid sequence with the predicted chicken LRP6 protein [29].

## 3. LRP6 Expression and Function during Development

Lrp6 expression has been detected in different tissue types in various species [5,30,31]. It plays a critical role during the development of vertebrates [2,4]. Lrp6 is involved in embryogenesis processes, in which it mediates the biological activity of canonical Wnt signalling [3,32]. For example, the complete deletion of Lrp6 in mice leads to several embryonic defects, and the mutant embryos die at birth, showing an important function for Lrp6 during embryogenesis in vertebrates [3,33]. Thus, we discuss here the expression and function of LRP6 in Xenopus, chicken, and mouse embryos.

In *Xenopus laevis*, Xlrp6 is maternally expressed in oocytes, and its transcripts have been detected in neural tissue and predominantly in the head regions, covering the branchial arches, eyes, and otic and olfactory placodes [5]. Xlrp6 expression was observed throughout the foregut mesoderm and endoderm [32]. It was able to activate Wnt–Fzd signalling and induced Wnt target genes to cause duplication of the dorsal axis [34]. This indicates an important role for Xlrp6 in Wnt activation and body patterning. Furthermore, Xlrp6 is involved in neural crest formation and axis patterning and in convergent extension movements [35,36]. Consistent with the above, the injection of lrp6 Morpholino or a dominant negative form leads to the inhibition of neural-crest formation, while lrp6 mRNA injection induces neural crest formation [33]. These results show a critical function for Xlrp6 in neural crest development.

Xlrp6 caused the formation of a partial second axis when it was overexpressed on the ventral side of the 4-cell-stage embryos [34]. In Xenopus, Lrp6 is important for Wnt11-activated dorsal axis formation [35]. Gain- or loss-of-function studies in Xenopus showed that Xlrp6 reduces convergent extension during embryogenesis; it is therefore considered to be an important regulator in this event [37]. Clearly, Xlrp6 function is required during the development of Xenopus embryos.

In chicken embryos, Lrp6 expression was reported in the developing lung and spinal cord tissues. In early stages of lung formation, Lrp6 transcripts are expressed in the border of lung and mesothelium [30]. In the developing spinal cord, Lrp6 was detected in the ventricular zone along the dorsal–ventral side of the neural tube at stage HH19, in the region of the precursors of dorsal interneurons, and in motoneurons at HH25 [36]. Lrp6 plays an important role during spinal cord development and transduces the activity of Wnt-FZD signalling [38]. For example, Lrp6 is required for commissural axon guidance in the developing spinal cord, as its downregulation by in ovo RNAi results in the disruption of axonal pathfinding at the floor plate of the neural tube [36], suggesting that canonical Wnt/LRP6 signalling is necessary for the correct guidance of postcrossing commissural axons.

In addition, Lrp6 is involved in neural tube patterning, since it interacts with Fzd10 to mediate Wnt1 activity in this tissue. A gain-of-function approach using in ovo electroporations showed that the co-transfection of Lrp6 and Fzd10 with Wnt1 led to an obvious increase in cell proliferation and induced the dorsal markers of the neural tube. Lrp6 with Fzd10 and Wnt1 enhanced the Wnt1-induced activation of luciferase expression (Wnt signalling reporter; top-flash assay) [38]. These observations indicate that Lrp6 is involved in mediation of canonical Wnt signalling activity during dorsoventral neural tube patterning, and in neural proliferation in the developing chick neural tube [38]. Thus, Lrp6 activity contributes to the development of the central nervous system in chick embryos.

In mice, Lrp6 is involved in gastrulation and plays an essential role in the early development of mouse embryos, as Lrp5/Lrp6 double-homozygous mutants fail to form a primitive streak [31]. Both receptors are required for the patterning of the posterior primitive ectoderm [31]. Moreover, embryos die shortly after gastrulation in Lrp5+/−; Lrp6−/− mutants [31]. This study suggested a functional redundancy between Lrp6 and Lrp5, but that Lrp6 plays a more essential role in development [11]. Importantly, total inactivation of Lrp6 led to multiple abnormalities in many organs, including the heart, eyes, limbs, brain, and developing spinal cord, and resulted in perinatal mortality [39,40]. This shows that Lrp6 has a critical and primary role in development.

Malformations of skeletal elements, osteoporosis, and spina bifida were observed in a spontaneous Lrp6rs allele mouse [39]. Lrp6 seems to mediate Wnt7a activity in the patterning of dorsal and posterior limbs during development in mice [41]. Consistent with this, in Wnt7a−/− Lrp6+/− double mutants, posterior skeletal elements were lost. Lrp6 plays important roles with Lrp5 in skeletal patterning and bone formation, as the deletion of these receptors specifically in the embryonic mesenchyme leads to an absence of osteoblasts in the embryo [41,42].

Lrp6 is required for spinal cord development, as it is expressed in the neural tube, and its mutation results in neural tube defects, including a failure of neural tube closure and disruption in cell polarity [39,43]. Genetic studies have revealed that both the loss and gain of Lrp6 function lead to a disruption of apical–basal cell polarity in the neural folds in Lrp6−/− mutants and Lrp6Cd/Cd embryos [43]. These phenotypes indicate that Lrp6 is required during the development of various tissue types including developing spinal cord in mammals, and its dysregulation leads to embryonic defects and malformations.

## 4. LRP6 Expression and Involvement in Human Diseases

LRP6 is ubiquitously expressed in human tissues, and it shows weak and strong expression patterns in various tissue types in humans [27]. Weak expression has been detected in a few tissue types, including the small intestine, skeletal muscle, liver, and thymus. The strongest expression has been reported in tissue types such as the heart, pancreas, brain, ovaries, and spleen. Furthermore, LRP6 expression was found in the placenta, breast, prostate, and colon [44] (Supplementary Figure S2). LRP6 expression could suggest important biological activities in these tissues through Wnt signalling. LRP6 is a co-receptor of Wnt/β-catenin signalling; therefore, it is required in the regulation of cell proliferation, specification, migration, and stem cell homeostasis [1,2].

Wnt signalling is involved in several complex human diseases, including cancer [45,46,47,48]. Deregulation of the Wnt signalling family members, including LRP6, has been reported to alter Wnt signalling activity and then lead to induction of cancer-associated genes as well as abnormal cell proliferation [49,50,51]. In humans, LRP6 mutations and its high expression have been detected in several types of cancers, including non-small-cell lung, bladder, breast, and colorectal cancers [52] (Supplementary Figure S3). For example, an LRP6 variant (rs6488507) in non-small-cell lung cancer (NSCLC) patients is linked with an increase in the risk of NSCLC in tobacco smokers [53]. A different LRP6 variant (rs10845498) is associated with decreased risk of lung squamous cell carcinoma.

Moreover, overexpression of LRP6 has been shown in breast cancer and its inhibition reduced breast cancer growth [52,54]. Three LRP6 missense variants were identified in patients with early colorectal cancer, and these variants were predicted to be pathogenic and to increase Wnt signalling activity in vitro [55]. In addition, LRP6 activity is required to induce cell proliferation in prostate cancer [56]. Consistent with these observations, in vivo cell proliferation and tumour growth of cancerous cells are reduced as a result of the inhibition of LRP6 expression [52]. Thus, LRP6 is being investigated as a biological indicator for assessment of the risk of some cancer types.

Furthermore, clinical studies and genomic analysis have confirmed that LRP6 genetic variants contribute to the progression of hypertension, atherosclerosis, hypercholesterolemia, and other diseases [24,57]. LRP6 mutations have been detected in Alzheimer’s disease, osteoporosis syndrome, and diabetes [57]. The disruption of LRP6 activities has been linked with several causes of heart disease, including increased serum LDL, glucose level, and triglycerides [24], indicating that LRP6 appears to be important for the regulation of glucose and lipid metabolism [58,59].

The deregulation of LRP6 function is involved in fatal cardiac arrhythmia in adulthood [60,61]. For example, a missense mutation of LRP6 was reported in patients with coronary artery disease linked with metabolic syndrome [57]. In addition, several LRP6 variants have been documented in patients with congenital tooth agenesis [62,63,64]. Recently, a novel single-nucleotide insertion in the LRP6 gene (c.1924dup) was identified via whole-exome sequence analysis in Japanese patients with tooth agenesis (oligodontia) [65]. These LRP6 variants indicate that LRP6 is required for proper development of human teeth, and further investigation could lead to a treatment for this disease.

## 5. LRP6 as a Potential Therapeutic Target

The involvement of LRP6 in human diseases has attracted researchers to investigate its potential as a therapeutic target for Wnt-related disorders [48,66]. Considerable efforts have led to the successful characterisation of some inhibitors against LRP6 and Wnt signalling, such as LRP6 antibodies [16,67], miR-27a [68], miR-126-3p [69], and SOST [70]. Further research is needed to develop specific inhibitor or activator drugs to target Lrp6. However, it is worth mentioning that some reports have suggested that LRP6 could interact with other pathways independently from Wnt/β-catenin and, in turn, regulate different biological functions [11,23]. For example, LRP6 could regulate the Wnt/PCP pathway and is implicated in cell polarity through RhoA activity [37,43]. LRP6 may be involved in Wnt/Ca+ pathway activation [36,71]. To achieve optimal parathyroid hormone (PTH) signalling activation, LRP6 may possibly act as a coreceptor for the PTH receptor [72]. Cardiomyocyte proliferation is regulated by LRP6 via the ING5/P21 pathway [73]. Therefore, further molecular and functional investigations are required to fully understand these possible LRP6 interactions, which must be taken into consideration when Lrp6 is targeted for a potential therapeutic purpose. In this context, the availability of the 3D structure of the HLRP6 protein (Figure 1B) and other genomic, biochemical, and molecular tools should be exploited to overcome these challenges, and to design molecules to target LRP6 activities in a specific and dependent manner that are selective and have minimal side effects.

## 6. Conclusions

LRP6 plays essential functions in the activation of Wnt/β-catenin signalling, which results in the regulation of numerous cellular processes, including cell proliferation and tissue homeostasis. LRP6 also controls embryonic development through different biological mechanisms in many living organisms including *Xenopus laevis*, chickens, mice, and humans. In contrary, impaired LRP6 signalling is linked with development defects and human diseases. Hence, more studies are needed to understand the complexity of LRP6 signalling during development and its pathological role in diseases, as it is able to activate different signalling pathways, leading to the regulation of cellular activities independent of canonical Wnt/β-catenin signalling. Furthermore, LRP6 is associated with complex diseases, such as hypercholesterolemia, atherosclerosis, Alzheimer’s disease, osteoporosis, heart diseases, diabetes, and cancers. Thus, targeting this receptor may offer very promising treatments for these diseases.

## Data Availability

Not applicable.

## Supplementary Materials

The following are available online at https://www.mdpi.com/article/10.3390/genes13010120/s1, Figure S1: This shows the alignment of human LRP6 sequence with the template sequence that used to create protein 3D structure; Figure S2: RNA expression of Human LRP6 in several human organs and tissues. This chart was obtained from Human Protein Atlas; Figure S3: Lrp6 RNA expression overview showing RNA-seq data from The Cancer Genome Atlas (TCGA). Lrp6 expression in 17 cancer types and categorized based on the mRNA expression level.

## Abbreviations

The following abbreviations are used in this manuscript:

APC	Adenomatous polyposis coli
CKI	Casein kinase I
DKK1	Dickkopf-related protein 1
Dvl	Dishevelled
EM	Electron microscopy
FZD	Frizzled
LDL	Low-density lipoprotein
LRP5/6	Low-density lipoprotein-related receptor protein
PTH	Parathyroid hormone
SOST	Sclerostin
Wnt	Wingless-type MMTV integration site
ZNRF3	Zinc and ring finger 3
