# Supplementary material for: LRP6 Receptor Plays Essential Functions in Development and Human Diseases
Source: Genes (Basel). 2022 Jan 10;13(1):120. doi: 10.3390/genes13010120 (PMC8775365; doi:10.3390/genes13010120)
Supplement: Supplementary file 1 [file genes-13-00120-s001.zip › genes-1517245-supplementary.pdf]

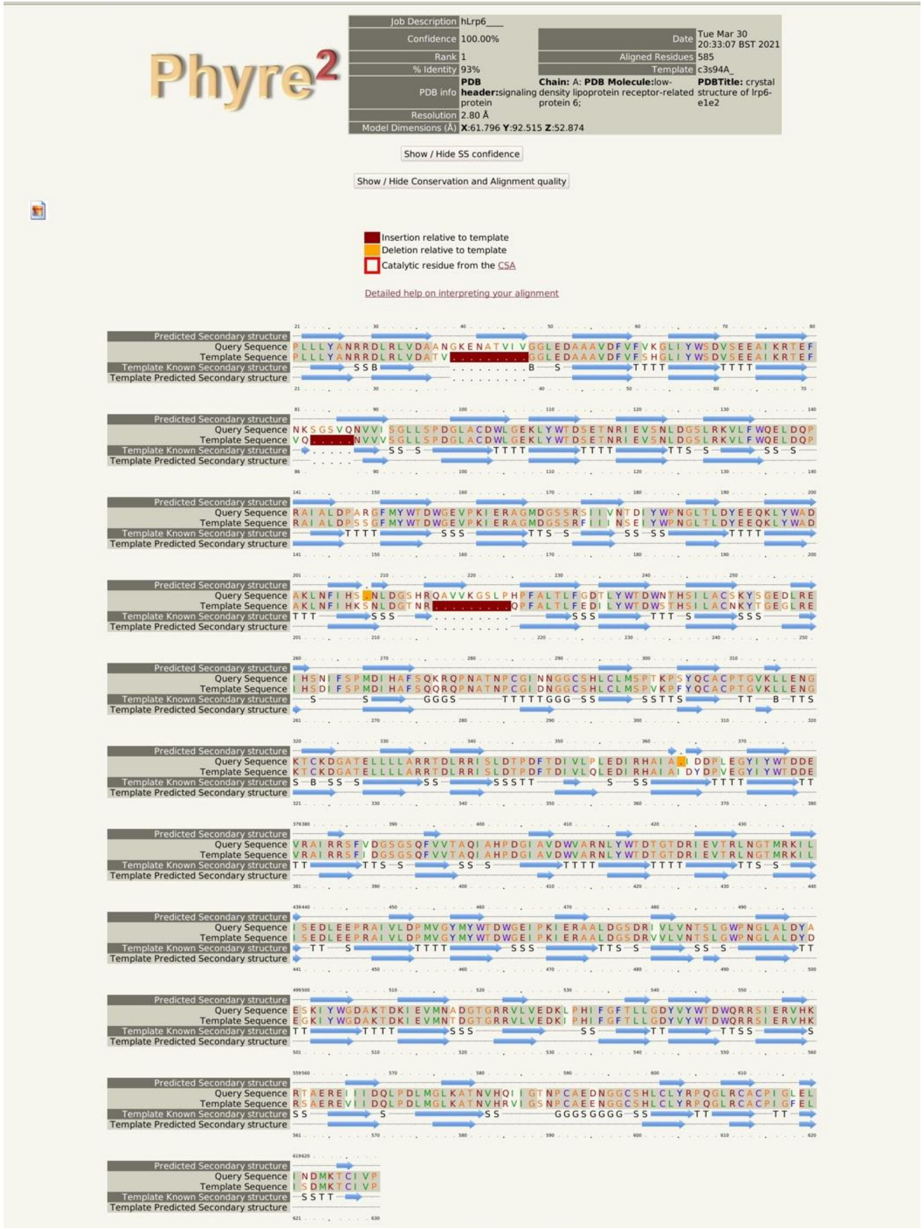

Figure S1. This shows the alignment of human LRP6 sequence with the template sequence that used to create protein 3D structure.

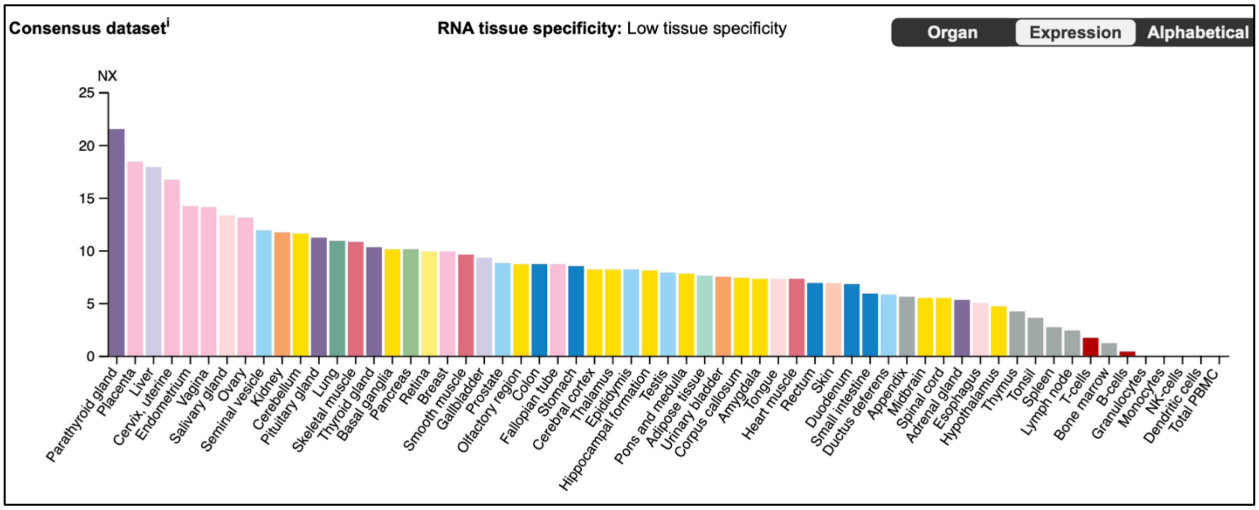

Figure S2. RNA expression of Human LRP6 in several human organs and tissues. This chart was obtained from Human Protein Atlas.

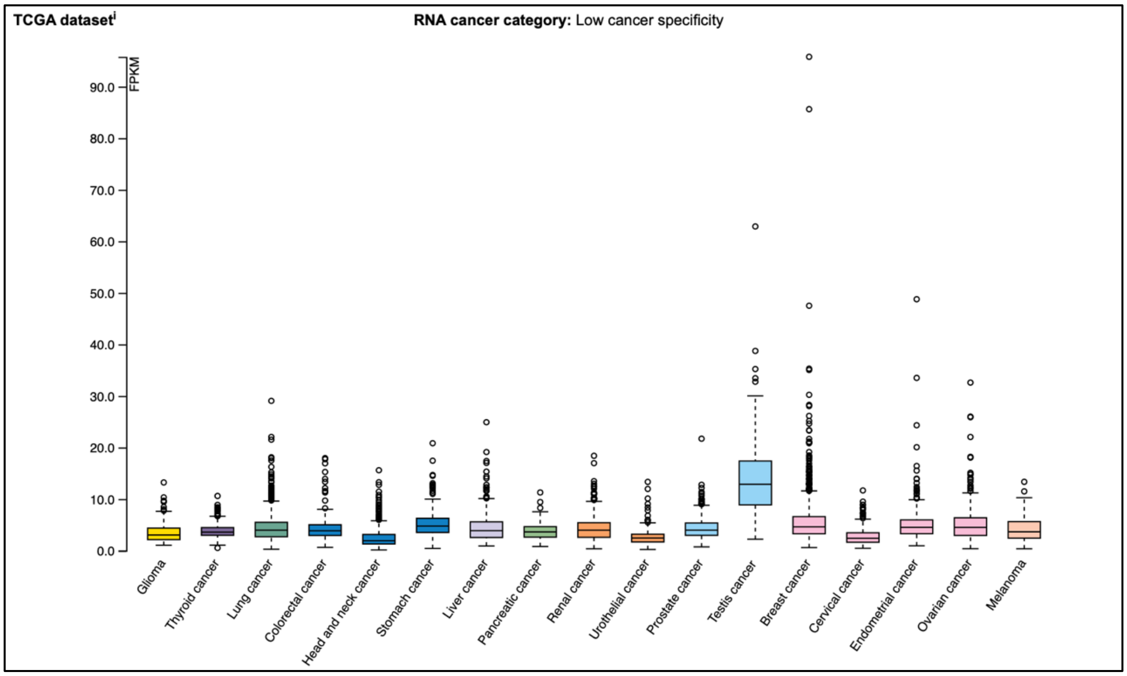

Figure S3. Lrp6 RNA expression overview showing RNA-seq data from The Cancer Genome Atlas (TCGA). Lrp6 expression in 17 cancer types and categorized based on the mRNA expression level.
